# Current clinical challenges and opportunities in oncologists' familiarity and understanding of immuno-oncology

**DOI:** 10.1186/2051-1426-2-S3-P180

**Published:** 2014-11-06

**Authors:** Tara Herrmann, Charlotte Warren

**Affiliations:** 1 Medscape Education, Fort Sam Houston, USA; 2 Medscape Education, New York, USA

## 

Advances in understanding of the immune system are changing the way oncologists treat cancer. In recent years, researchers have increasingly looked to harnessing the patient's immune system in the management of difficult to treat cancers. While such an approach was once felt to be unattainable, a better understanding of how the adaptive immune system works has allowed the field of immuno-oncology to move to the clinic with increasing success. This study's objective was to assess practicing oncologists' familiarity with, and understanding of, immuno-oncology concepts that will play an important role in the care of patients with metastatic cancer such as those with lung cancer.

An expert panel was convened to identify knowledge gaps in the area of immuno-oncology. A series of 9 CME activities are planned, 4 of which have been developed and posted online. Interactivity questions allowed learners to self-report their familiarity with and/or confidence in concepts related to immuno-oncology. The outcomes survey consisted of knowledge-based questions that examined knowledge of the evidence-base surrounding immuno-oncology. Confidentiality of survey respondents was maintained and responses were de-identified and aggregated prior to all analyses.

A total of 469 of oncologist have participated in the surveys. Overall, oncologists reported a lack of familiarity and need for additional education in the areas of immunology as well as implications for immuno-oncology agents. Less than 15% of oncologists reported being very familiar with the immune system while 72% of learners indicated a need for additional education on adaptive immune responses. In addition, only 7% and11% of oncologists reported being very familiar with the concepts of immune escape and check points, respectively. Over 60% of oncologists reported little familiarity with emerging immunotherapies for the treatment of metastatic solid tumors, and a majority did not have a firm understanding of how to assess a patient's response. In alignment with learners' self-assessment, pre-education testing indicated knowledge and practice gaps in understanding of T cell regulation as well as mechanisms of immune escape, MOA of immune checkpoint inhibitors, understanding of clinical efficacy data, and assessing treatment response. Finally, initial analysis indicated that oncologists' participation in these CME activities had an impact on their knowledge and competence.

This study identified knowledge, competency, and practice gaps in the understanding and use of immune checkpoint inhibitors in metastatic cancer. Further assessment of oncologists' responses after participating in the remaining educational intervention set is planned to demonstrate improvement in oncologists understanding and clinical practice.

